# The NKG2D – IL-15 signaling pathway contributes to T-cell mediated pathology in inflammatory myopathies

**DOI:** 10.18632/oncotarget.6462

**Published:** 2015-12-04

**Authors:** Tobias Ruck, Stefan Bittner, Ali Maisam Afzali, Kerstin Göbel, Sarah Glumm, Peter Kraft, Claudia Sommer, Christoph Kleinschnitz, Corinna Preuβe, Werner Stenzel, Heinz Wiendl, Sven G. Meuth

**Affiliations:** ^1^ Department of Neurology, University of Muenster, Muenster, Germany; ^2^ Department of Neurology, University Medical Center of the Johannes Gutenberg-University Mainz, Mainz, Germany; ^3^ Department of Neurology, University Hospital of Wuerzburg, Wuerzburg, Germany; ^4^ Department of Neuropathology, Charité-Universitätsmedizin, Berlin, Germany

**Keywords:** NKG2D, IL-15, polymyositis, idiopathic inflammatory myopathies, T cell activation, Pathology Section

## Abstract

NKG2D is an activating receptor on T cells, which has been implicated in the pathogenesis of autoimmune diseases. T cells are critically involved in idiopathic inflammatory myopathies (IIM) and have been proposed as specific therapeutic targets. However, the mechanisms underlying T cell-mediated progressive muscle destruction in IIM remain to be elucidated. We here determined the involvement of the NKG2D – IL-15 signaling pathway. Primary human myoblasts expressed NKG2D ligands, which were further upregulated upon inflammatory stimuli. In parallel, shedding of the soluble NKG2D ligand MICA (sMICA) decreased upon inflammation potentially diminishing inhibition of NKG2D signaling. Membrane-related expression of IL-15 by myoblasts induced differentiation of naïve CD8^+^ T cells into highly activated, cytotoxic CD8^+^NKG2D^high^ T cells demonstrating NKG2D-dependent lysis of myoblasts *in vitro*. CD8^+^NKG2D^high^ T cell frequencies were increased in the peripheral blood of polymyositis (PM) patients and correlated with serum creatinine kinase concentrations, while serum sMICA levels were not significantly changed. In muscle biopsy specimens from PM patients expression of the NKG2D ligand MICA/B was upregulated, IL-15 was expressed by muscle cells, CD68^+^ macrophages as well as CD4^+^ T cells, and CD8^+^NKG2D^+^ cells were frequently detected within inflammatory infiltrates arguing for a local signaling circuit in the inflammatory muscle milieu. In conclusion, the NKG2D – IL-15 signaling pathway contributes to progressive muscle destruction in IIM potentially opening new therapeutic avenues.

## INTRODUCTION

Idiopathic inflammatory myopathies are a heterogeneous group of muscle disorders characterized by chronic muscle inflammation and progressive muscle weakness. Polymyositis (PM), dermatomyositis (DM) and inclusion body myositis (IBM) are the three major subsets based on distinct clinical and histopathological features [1, 2]. The classical view is that CD8^+^ T cells are more common in PM and IBM, while CD4^+^ T cells are predominantly found in DM. Indeed, in PM, muscle cell injury appears to be mediated directly by CD8^+^ T cells that surround and invade MHC-I expressing myofibers [3, 4]. However, new insights into the pathogenesis of idiopathic inflammatory myopathies have identified muscle-resident CD4^+^ and CD8^+^ T cells in PM, DM and IBM as predominantly CD28^null^ T cells. CD4^+^CD28^null^ and CD8^+^CD28^null^ T cells are terminally differentiated apoptosis-resistant subsets which produce high levels of cytokines (mainly IFNγ and TNFα), contain perforin and granzyme B and have a high cytotoxic potential [5-7]. Especially in the case of CD4^+^CD28^null^ cells, the fundamental differences to conventional CD4^+^ T helper cells are obvious and underline the need to reevaluate the local immunological milieu in the inflamed muscle tissue that drives and maintains T cell functions [8]. In this context, muscle cells and myoblasts actively shape (auto)immune reactions as they secrete inflammatory cytokines and chemokines or express costimulatory molecules [9-11].

The activating receptor NKG2D (natural-killer group 2, member D, CD314) is mainly expressed on NK cells, CD8^+^ T cells and γδ T cells, but also on small specialized immune-cell subsets (e.g. CD4^+^NKG2D^+^ T cells [12, 13]) that acquire cytotoxic effector functions [14, 15]. The function of NKG2D on CD8^+^ T cells has been initially described as a co-stimulatory signal supporting T cell activation after T cell receptor (TCR) stimulation [16, 17]. Under certain conditions, however, CD8^+^ T cells acquire the capacity to directly target cells via NKG2D without any contribution of the TCR [18-20]. Chronic activation *in vitro* or in autoimmune conditions in patients suffering from celiac disease [20, 21] have been shown to drive CD8^+^ T cells towards this effective cytolytic phenotype which has sometimes also been included in the heterogenous group of so-called “lymphokine activated killer cells” [22, 23]. Chronic stimulation via the IL-15 signaling pathway has been implicated as key mechanism determining the ability of NKG2D to act as a TCR-independent stimulatory molecule on tissue-resident cytolytic CD8^+^ T cells [20, 24].

Ligands for NKG2D (MICA/B (MHC class I chain-related protein A and B) and the UL16 binding proteins (ULBP1-6) are rarely detectable on healthy tissues and their expression seem to be tightly controlled [15, 25, 26]. However, they are frequently upregulated upon cellular stress signals like viral infections, tumorgenesis or inflammation rendering cells susceptible to NKG2D-mediated cytotoxicity [20]. Alternatively, NKG2D ligands are involved in immunosuppressive pathways. Metalloproteases are known to release MICA (soluble MICA, sMICA) and other NKG2D ligands from the cell surface resulting in a downregulation of NKG2D expression on CD8^+^ T cells which has been demonstrated as a route of immune evasion of tumor cells [27, 28].

The NKG2D signaling pathway has already been implicated in other autoimmune disorders such as rheumatoid arthritis, giant cell arteritis, polymyalgia rheumatica, multiple sclerosis or Crohn's disease [13, 29-32]. Our study investigated the putative role of NKG2D – IL-15 signaling for CD8^+^ T cell mediated pathology in inflammatory myopathies.

## RESULTS

### NKG2D ligands are upregulated on primary human myoblasts under inflammatory conditions

**Figure 1:**
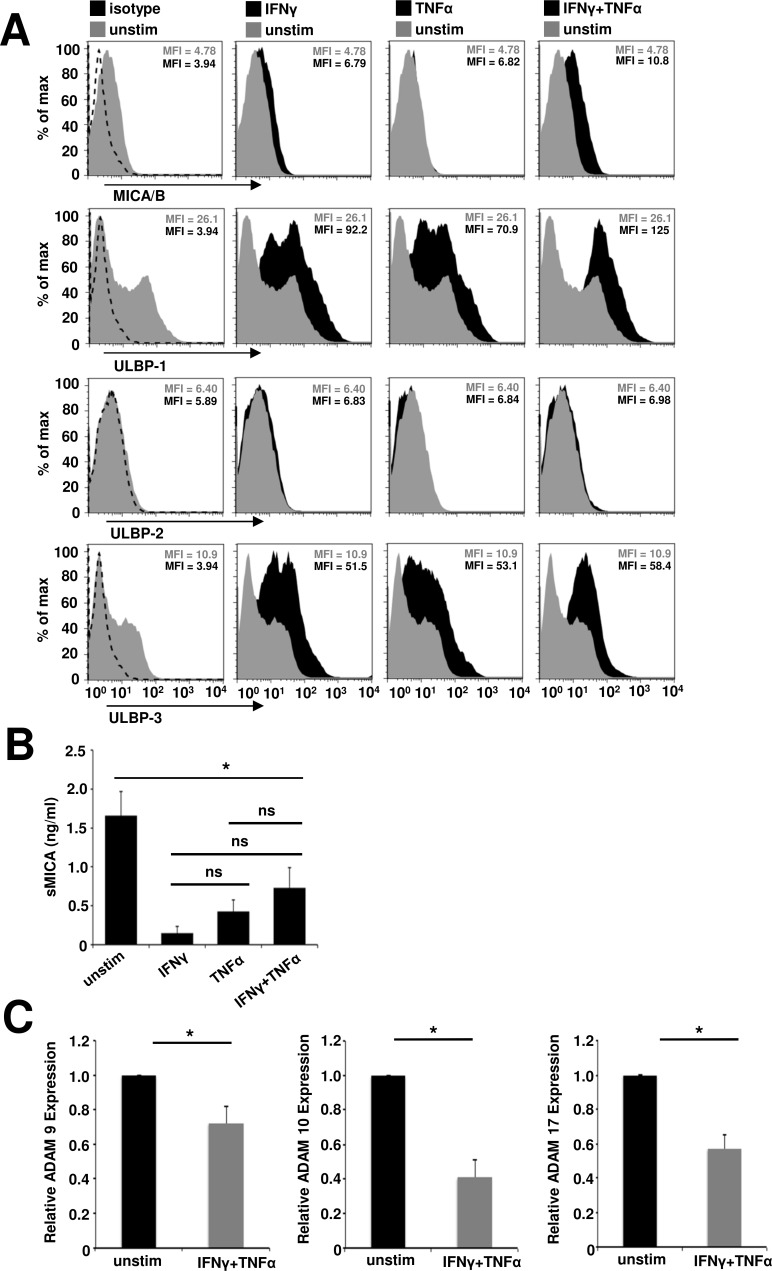
Inflammation of primary human myoblasts results in an upregulation of surface expression, but reduced shedding of NKG2D ligands **A.** The surface expression of NKG2D ligands on primary human myoblasts was assessed under different inflammatory conditions (IFNγ: 1000 U/ml and/or TNFα: 1000 U/ml for 48 h). Histograms show the fluorescence intensity for the NKG2D ligands of unstimulated (grey, unstim) and inflamed (black) myoblasts or the isotype control (dashed line), one representative example is shown (n = 5) and mean fluorescence intensity (MFI) of each population is depicted. **B.** Soluble MICA (sMICA) ELISA of human myoblast cell culture supernatants. Myoblasts were treated with IFNγ (1000 U/ml) and/or TNFα (1000 U/ml) for 48 h (n = 4). **C.** Relative expression of ADAM (A Disintegrin and Metalloproteinase) peptidase proteins 9, 10 and 17, responsible for NKG2D ligand shedding, under basal and inflammatory conditions in human myoblasts assessed by RT-PCR (n = 4). * *p* < 0.05, ns = not significant.

NKG2D ligands are induced by cellular stress and have been shown to mediate NKG2D-dependent, cell-type specific pathology in several autoimmune diseases [33]. As a prerequisite for muscle cell-specific, NKG2D-dependent pathology in inflammatory myopathies we investigated the NKG2D ligand expression on primary human myoblasts under basal and inflammatory conditions. Highly enriched primary human myoblast cell cultures (purity > 98%, Suppl. Figure 1) expressed the NKG2D ligands MICA/B, ULBP-1 and ULBP-3, which were found upregulated upon inflammation. However, there was no ULBP-2 expression (Figure 1A). Highest expression levels of these ligands were observed under combined IFNγ and TNFα stimulation. In parallel, we observed significantly reduced levels of NKG2D-inhibitory, soluble MICA (sMICA) in the cell culture supernatant under inflammatory conditions (basal conditions: 1.66 ± 0.31 ng/ml, IFNγ: 0.15 ± 0.1 ng/ml, TNFα: 0.43 ± 0.15 ng/ml, IFNγ plus TNFα: 0.73 ± 0.26 ng/ml, Figure 1B). However, there were no significant differences among the inflammatory conditions. In accordance, we found a significant downregulation of NKG2D ligand shedding ADAMs (A Disintegrin and Metalloproteinase) 9, 10 and 17 [34] in human myoblasts by IFNγ plus TNFα treatment (Figure 1C) corroborating previous findings demonstrating diminished ADAM9, ADAM10, ADAM17 and ADAM19 gene expression in myoblasts under pro-inflammatory stimuli *in vitro* [35].

### Sustained IL-15 stimulation converts naïve CD8^+^ T cells into CD8^+^NKG2D^high^ highly activated, cytotoxic effector T cells *in vitro*

**Figure 2:**
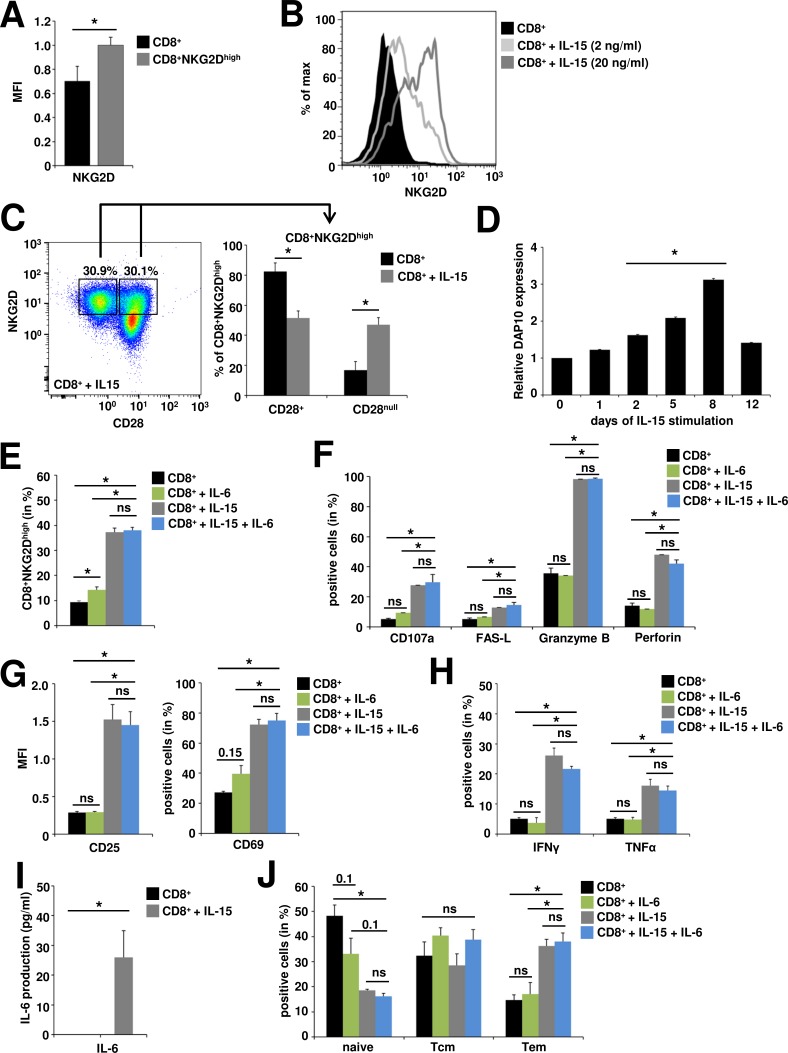
*In vitro* generated CD8^**+**^NKG2D^**high**^ cells are highly activated, cytotoxic effector T cells CD8^+^ T cells were pre-activated with anti-CD3 (plate-bound, 1 μg/ml, 24 h) and stimulated with IL-6 (1 ng/ml, 8 d), IL-15 (20 ng/ml, 8 d) or IL-6 and IL-15 as indicated. **A.** IL-15 stimulated CD8^+^ T cells show upregulated surface expression of NKG2D (CD8^+^NKG2D^high^) as assessed by flow cytometry (n = 3). **B.** NKG2D expression on CD8^+^ T cells following IL-15 stimulation. **C.** Proportions of CD28^+^ and CD28^null^ cells within the CD8^+^NKG2D^high^ cell population under basal conditions and after IL-15 stimulation. Left panel: exemplary staining and gating strategy, right panel: quantitative evaluation (n = 9) **D.** DAP10 gene expression under long-term IL-15 stimulation, d0 = *ex vivo* expression (n = 10). **E.** CD8^+^NKG2D^high^ cells are generated by IL-15 and to much lesser extents by IL-6 stimulation, with no synergistic effects for the combination of IL-15 and IL-6. Only IL-15 generated CD8^+^NKG2D^high^ cells express high levels of markers indicative for cytotoxic propensity (**F.**, n = 6) and activation (**G.**, n = 3), produce high amounts of the pro-inflammatory cytokines IFNγ and TNFα (**H.**: flow cytometry, n = 6), low but detectable levels of IL-6 (I: ELISA, n = 6) and are skewed to an effector memory phenotype (J; Tem, T effector memory, CD44^+^CD62L^−^; Tcm, T central memory, CD44^+^CD62L^+^; naïve, naïve T cells CD44^−^CD62L^+^) (n = 3). * *p* < 0.05, ns = not significant.

IL-15 promotes distinct changes in the NKG2D signaling pathway arming TCR-independent cytolytic responses of CD8^+^ T cells [20]. In accordance with previous reports, we observed a strong (Figure 2A) and dose-dependent (Figure 2B) upregulation of NKG2D following stimulation with recombinant IL-15, generating a CD8^+^NKG2D^high^ T cell population with highest frequencies at day 8 after stimulation (Suppl. Figure 2A). Muscle-resident CD8^+^ T cells in idiopathic inflammatory myopathies are predominantly CD28 negative (CD8^+^CD28^null^) [5-7] and IL-15 has been implicated in their generation [36, 37]. Under chronic IL-15 stimulation the proportion of CD28^null^ cells within the CD8^+^NKG2D^high^ T cell population was significantly increased compared to control conditions (from 16.8% ± 5.7% to 47.1% ± 4.7%), however 51.4% ± 4.8% still displayed CD28 expression (Figure 2C). In parallel to NKG2D, the expression of DAP10, the intracellular signal transduction protein for NKG2D, was significantly increased under IL-15 stimulation in a time-dependent manner (Figure 2D), beginning at day 2 (fold-increase = 1.6, p = 0.02) and peaking at day 8 (fold-increase = 3.1, p = 0.00005). DAP10 expression has previously been shown to correlate with enhanced cytotoxicity of CD8^+^ T cells [19]. Accordingly, IL-15 generated CD8^+^NKG2D^high^ cells expressed significantly higher levels of CD107a (a degranulation marker, also known as LAMP-1), perforin, granzyme B and FAS-ligand (FAS-L) indicating a strong cytotoxic propensity (Figure 2F). Moreover, IL-15 generated CD8^+^NKG2D^high^ cells demonstrated a high activation status as assessed by CD25 and CD69 expression (Figure 2G) and produced high amounts of the pro-inflammatory cytokines IFNγ und TNFα (Figure 2H and Suppl. Figure 2B) and low but detectable levels of IL-6 (Figure 2I). Accordingly, the CD8^+^NKG2D^high^ population was skewed to an effector memory phenotype (CD8^+^ T cells: 17.7% ± 6%, CD8^+^NKG2D^high^: 34.1% ± 3.4%, n = 3, p = 0.02) thereby enabling immediate effector functions (Figure 2J). In a previous report, IL-6 has been demonstrated to show synergistic effects to IL-15 in the TCR-independent stimulation of proliferation and functional differentiation of CD8^+^ T cells [38]. However, IL-6 alone was neither able to generate as high frequencies nor to reproduce the phenotype of IL-15 generated CD8^+^NKG2D^high^ cells. In combination with IL-15 no additive effects were observed for IL-6 concerning cytotoxic propensity, activation status, cytokine production or memory phenotype of CD8^+^NKG2D^high^ cells (Figure 2E-2I).

### Myoblast derived IL-15 induces the generation of cytotoxic CD8^+^NKG2D^high^ T cells in *in vitro* coculture systems

**Figure 3:**
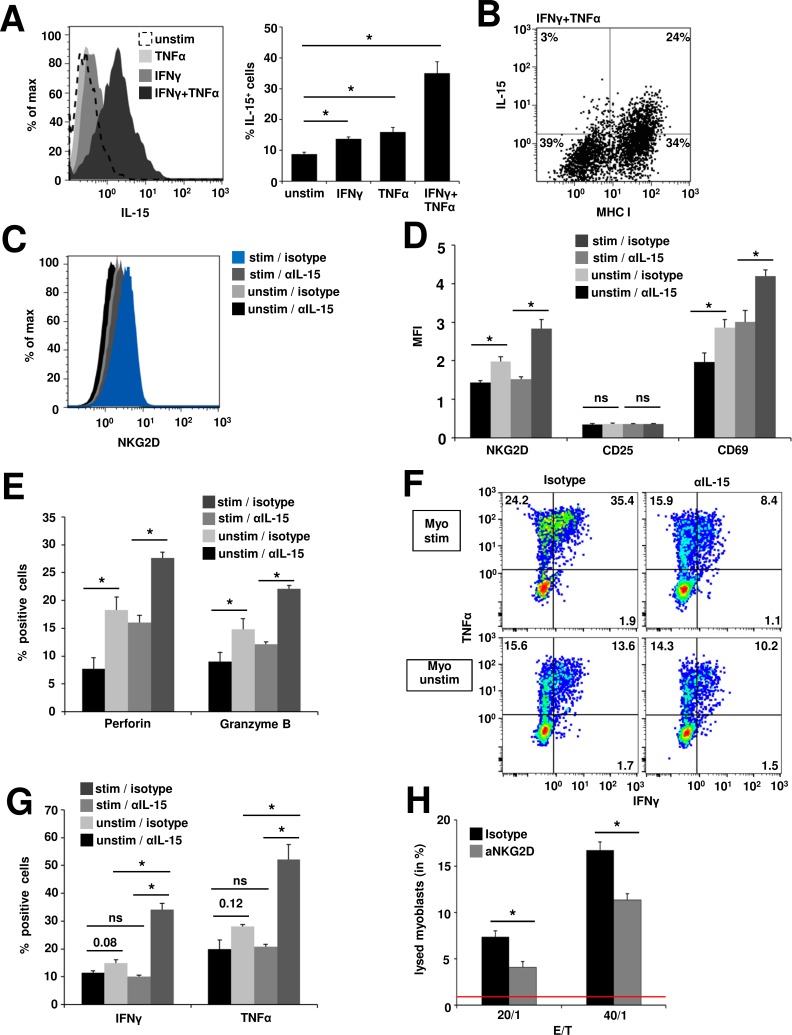
IL-15-dependent generation of cytotoxic CD8^**+**^NKG2D^**high**^ T cells in cocultures with inflamed human myoblasts **A.** Flow cytometry analysis of IL-15 surface expression on unstimulated and inflamed (IFNγ 1000 U/ml and/or TNFα 1000 U/ml, 48 h) human myoblasts. Left: Histogram depicting fluorescence intensity, right: percentage of IL-15^+^ myoblasts under different conditions. (n = 4) **B.** IL-15 expression positively correlates with MHC-I expression on IFNγ and TNFα treated myoblasts. A representative example is shown (n = 4) **C.** NKG2D expression on CD8^+^ T cells after 48 h coculture with unstimulated (unstim) or inflamed myoblasts (stim, IFNγ 1000 U/ml and TNFα 1000 U/ml, 48 h prior to coculture) in the presence of an IL-15 blocking antibody or isotype control (n = 4). IL-15-mediated upregulation of NKG2D is associated with increased T cell activation **D.**, perforin and granzyme B expression (**E.** and pro-inflammatory cytokine production (**F.** + **G.**) (n = 4). **H.** 5 h FATAL assay of human inflamed (IFNγ: 1000 U/ml and TNFα: 1000 U/ml for 48 h prior to coculture), DiD (membrane dye) / CFSE (cytosolic dye) stained myoblasts and CD8^+^NKG2D^high^ T cells (pretreated and isolated from myoblast cocultures as described in C). Blocking anti-NKG2D antibody (10 μg/ml) or the respective isotype control (10 μg/ml) was added to the coculture 4 h prior and during the assay. Bar graphs depict the proportion of lysed myoblasts (DiD^+^CFSE^−^) (n = 4). Spontaneous lysis of myoblasts is indicated by the red line (0.87% ± 0.46%).* *p* < 0.05, ns = not significant, E:T = effector/target ratio.

IL-15 exerts its signaling functions to neighbouring cells mainly in its surface-bound form [39]. Thus, to determine whether myoblasts cells are a relevant source of IL-15, we assessed the presence of surface IL-15 on human myoblasts. Under basal conditions only 8.7% ± 0.6% of myoblasts expressed IL-15. IFNγ or TNFα treatment slightly increased the proportion of IL-15^+^ cells (13.7% ± 0.7%, p = 0.01 or 15.9% ± 1.5%, p = 0.04 respectively), while combined application of IFNγ and TNFα resulted in an IL-15 expression in 35.1% ± 3.7% of all myoblasts (p = 0.007) (Figure 3A). IL-15 expression positively correlated with MHC-I expression on IFNγ and TNFα treated myoblasts (Figure 3B). However, IL-15 ELISA of myoblast cell culture supernatants showed neither under basal nor inflammatory conditions IL-15 concentrations exceeding detection limits (data not shown). In order to evaluate whether the effects of recombinant IL-15 can be reproduced by IL-15 dependent interactions between muscle cells and CD8^+^ T cells we conducted coculture experiments. Myoblasts were left unstimulated or inflamed with IFNγ and TNFα to yield high numbers of IL-15^+^ myoblasts. Then naive CD8^+^ T cells were cocultured in the presence of an IL-15 blocking antibody or isotype control for 48 h. In the presence of the blocking IL-15 antibody CD8^+^ T cells expressed significantly lower amounts of NKG2D compared to isotype control after coculture with unstimulated myoblasts (mean fluorescence intensity, MFI: 1.4 ± 0.1 vs. MFI 2.0 ± 0.1; p = 0.03). Cocultivation with inflamed myoblasts in the presence of the isotype control led to the highest upregulation of NKG2D (2.8 ± 0.2), while treatment with blocking IL-15 reduced NKG2D expression to levels found on unstimulated myoblasts (MFI 1.5 ± 0.1; p = 0.02) (Figure 3C + Figure 3D). Similar results were obtained for the expression of the activation marker CD69 (unstimulated myoblasts: anti-IL-15: MFI 2.0 ± 0.2 vs. isotype: MFI 2.9 ± 0.2, p = 0.049; inflamed myoblasts: anti-IL-15: MFI 3.0 ± 0.3 vs. isotype: MFI 4.2 ± 0.2, p = 0.03) (Figure 3D) and for the cytolytic proteins perforin (unstimulated myoblasts: anti-IL-15: MFI 7.7 ± 2.0 vs. isotype: MFI 18.3 ± 2.3, p = 0.03; inflamed myoblasts: anti-IL-15: MFI 16.0 ± 1.3 vs. isotype: MFI 27.6 ± 1.0, p = 0.001) and granzyme B (unstimulated myoblasts: anti-IL-15: MFI 8.0 ± 1.0 vs. isotype: MFI 14.8 ± 1.9, p = 0.047; inflamed myoblasts: anti-IL-15: MFI 12.2 ± 0.4 vs. isotype: MFI 22.1 ± 0.6, p = 0.0001) (Figure 3E). However, the expression of the intermediate activation marker CD25 remained unchanged in coculture with myoblasts, potentially due to short term stimulation (Figure 3D). Moreover, IL-15 blockade resulted in a significant reduction of IFNγ and TNFα production after cocultures with inflamed myoblasts (IFNγ: anti-IL-15: 10.1% ± 0.5% vs. isotype: 34.1% ± 2.3%, p = 0.002; TNFα: anti-IL-15: 20.8% ± 0.9% vs. isotype: 52.2% ± 5.4%, p = 0.01). However, the effects were less pronounced in cocultures with unstimulated myoblasts (IFNγ: anti-IL-15: 11.4% ± 0.7% vs. isotype: 14.9% ± 1.2%, p = 0.08; anti-IL-15: TNFα = 19.9% ± 3.3% vs. isotype: 28.1% ± 0.6%, p = 0.12) (Figure 3F + Figure 3G). To determine whether the increased NKG2D expression and content of lytic enzymes in CD8^+^ T cells results in augmented cytotoxic activity towards myoblasts, we used a FATAL assay. Inflamed human myoblasts (inflamed with IFNγ and TNFα for 48 h prior to coculture) and CD8^+^NKG2D^high^ T cells (pretreated and isolated from myoblast cocultures as described in C) were cocultured for 5 h. Treatment with a blocking anti-NKG2D antibody significantly decreased the proportion of lysed myoblasts compared to isotype control. The proportions of lysed myoblasts increased with higher effector/target ratios (E/T = 20:1: 4.1% ± 0.6% vs 7.4% ± 0.6%, p = 0.049; E/T = 40:1: 11.4% ± 0.7% vs. 16.7% ± 0.9%, p = 0.02; spontaneous lysis of myoblasts = 0.87% ± 0.46%) (Figure 3H). Thus, IL-15 produced by human myoblasts generates highly activated, cytotoxic effector CD8^+^NKG2D^high^ T cells in the inflamed muscle during inflammatory myopathies thereby contributing to progressive muscle destruction.

### Alterations in the NKG2D signaling pathway are present in the muscle and peripheral blood of polymyositis patients

**Figure 4:**
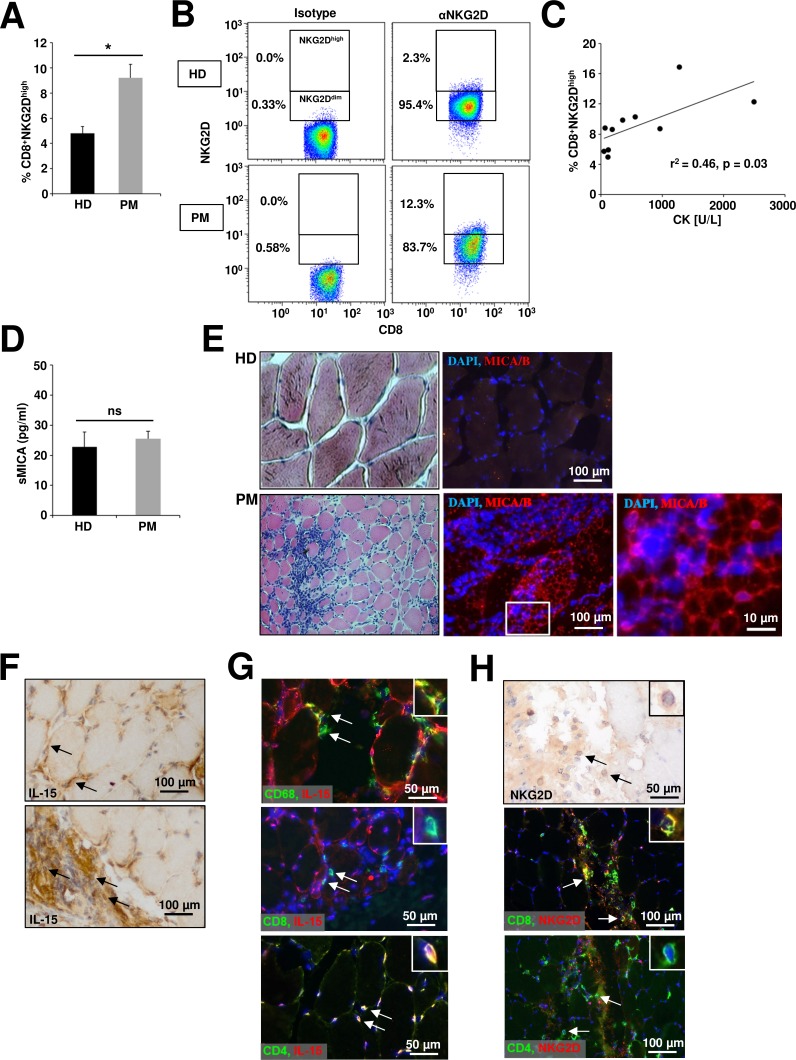
Alterations of the NKG2D – IL-15 signaling pathway in peripheral blood and muscle biopsies of polymyositis patients **A.** Flow cytometry analysis of CD8^+^ T cells (PBMCs) from healthy donors (HD, n = 13) and polymyositis patients (PM, n = 10) stained for NKG2D. Cells were gated for CD8^+^NKG2D^dim^ and CD8^+^NKG2D^high^ subsets as depicted in **B. C.** Correlation of CD8^+^NKG2D^high^ percentages in the peripheral blood of PM patients with serum creatinine levels at the same time-point **D.** Concentration of the soluble NKG2D ligand MICA assessed by ELISA in serum of HD and PM patients (n = 5). **E.** Histopathological characterization of representative human muscle biopsy specimen from a healthy donor (HD, n = 6) and a PM patient (n = 6) Left panel: hematoxylin/eosin (HE) staining. Right panel: DAPI (blue) and anti-MICA/B (red). Scale bars represent 100 μm or 10 μm respectively. **F.** IL-15 is expressed by muscle cells (predominantly membrane-bound, upper panel) and muscle-infiltrating immune cells (lower panel) in biopsy specimens of PM patients. IL-15 expression was detected by HRP-DAB staining and tissue was counterstained with haemalum (n = 3). **G.** Coexpression of IL-15 (red) and CD68 (green, upper panel), CD8 (green, mid panel) or CD4 (green, lower panel) on muscle-infiltrating immune cells (n = 3) in PM. Nuclei are stained with DAPI (blue). **H.** Muscle-infiltrating immune cells with lymphocytic morphology express NKG2D (HRP-DAB staining and counterstaining with haemalum; NKG2D^+^ (immunofluorescence: red) cells are positive for CD8 (immunofluorescence: green) and negative for CD4 (immunofluorescence: green). * *p* < 0.05, ns = not significant.

In PM CD8^+^ T cells are critically involved in the progressive destruction of muscle cells [40]. Moreover, muscle cells and infiltrating macrophages in PM are a prominent source of IL-15 potentially arming NKG2D-dependent pathophysiological mechanisms [41, 42]. Hence, we chose PM as a prototypic inflammatory myopathy to investigate whether our *in vitro* data can be translated into the *in vivo* situation. Flow cytometry analysis of peripheral blood mononuclear cells from PM patients revealed a significant accumulation of CD8^+^NKG2D^high^ T cells (Figure 4A + Figure 4B) compared to healthy donors (HD) (9.2 % ± 0.5 vs. 4.8% ± 0.6, p = 0.003), while no differences were found for various other immune cell subtypes (Suppl. Figure 3). The frequency of CD8^+^NKG2D^high^ T cells in peripheral blood positively correlated with serum CK levels as surrogate marker for disease activity (Figure 4C, r^2^ = 0.46, p = 0.03 + Table 1). However, we observed no significant differences for the soluble MICA concentration in the serum of healthy donors and PM patients (Figure 4D). In histopathological stainings from muscle biopsies only specimen from PM patients, but not from healthy donors showed expression of the NKG2D ligand MICA/B (Figure 4E). IL-15 was expressed by muscle cells predominantly at the cell-surface but also in the cytoplasm (Figure 4F, upper panel) and muscle-infiltrating immune cells (Figure 4F, lower panel). Within the muscle-infiltrating cells, CD4^+^ T cells, and a conspicuous proportion of CD68^+^ cells were stained positively for IL-15, whereas CD8^+^ T cells were IL-15 negative (Figure 4G). However, only CD8^+^ T cells but not CD4^+^ T cells expressed NKG2D in muscle biopsy specimens of PM patients (Figure 4H).

**Table 1:** Clinical and cellular characteristics of polymyositis patients

Patient	Sex	Age (years)	Disease duration (months)	Treatment	CK [U/L]	Frequency of CD8^+^NKG2D^high^ (%)
PM1	M	55	15	IVIG	2493	12,3
PM2	M	43	11	Pred	112	4,97
PM3	M	64	144	MTX + IVIG	560	10,3
PM4	F	57	5	IVIG	69	8,82
PM5	M	30	3	CP	356	9,85
PM6	M	44	6	Pred	183	8,62
PM7	M	56	19	Aza	53	5,73
PM8	F	65	4	Aza	961	8,74
PM9	F	63	13	Pred	1279	16,9
PM10	F	63	11	MTX	119	5,91

Patients with polymyositis (PM) were investigated for the frequencies of CD8^+^NKG2D^high^ T cells in peripheral blood and CK (creatinine kinase) levels in the serum.

Aza, azathioprine; CP, cyclophosphamide; IVIG, intravenous immunoglobulins; F, female; M, male; MTX, methotrexate; Pred, prednisolone.

## DISCUSSION

Our study demonstrates that human myoblasts upregulate NKG2D ligands upon inflammation *in vitro* rendering cells susceptible to NKG2D-mediated cell lysis. In an inflammatory milieu myoblasts express sufficient surface IL-15 so as to generate highly effective cytotoxic T cells capable of TCR-independent NKG2D-mediated killing, here called CD8^+^NKG2D^high^ T cells [20]. In agreement with a pathogenic role in idiopathic inflammatory myopathies, CD8^+^NKG2D^high^ T cell frequencies are significantly increased in peripheral blood of PM patients compared to healthy controls and CD8 / NKG2D double positive cells are highly prevalent in inflammatory infiltrates in PM muscle. Moreover, our *in situ* data document an upregulation of NKG2D ligands and IL-15 on muscle cells in biopsy specimens from PM patients indicating susceptibility to NKG2D and IL15-mediated immune responses.

Ligands for the activating receptor NKG2D belong to an abundant family of danger signals indicating cellular stress in the broadest sense. An inappropriate triggering or perpetuation of these danger signal pathways has been implicated in autoimmune processes like multiple sclerosis, rheumatoid arthritis and Crohn's disease such as intervention has shown beneficial effects in their respective disease models [12, 13, 29-31, 43, 44]. In our *in vitro* studies, the highest expression of NKG2D ligands was observed under combined IFNγ and TNFα treatment, two cytokines highly abundant in the muscle microenvironment of idiopathic inflammatory myopathies [40]. A comparable upregulation of NKG2D ligands was found in muscle biopsies of PM patients, which extend our previous findings [45].

NKG2D-mediated effector mechanisms of tissue-resident cytolytic CD8^+^ T cells are highly interdependent with the IL-15 signaling pathway [20, 24]. In our study, CD8^+^ T cells differentiated into highly effective cytotoxic CD8^+^NKG2D^high^ T cells capable of TCR-independent NKG2D-mediated killing upon chronic IL-15 stimulation [20]. In combination with IL-15, IL-6 has been previously reported providing synergistic effects for T cell proliferation albeit not significantly contributing to phenotypic T cell differentiation [38], a finding corroborated by our study demonstrating no relevant phenotypic changes to CD8^+^NKG2D^high^ cells by simultaneous IL-6 and IL-15 stimulation. However, CD8^+^NKG2D^high^ cells produced low but detectable levels of IL-6, which together with IFNγ and TNFα might sustain the proinflammatory milieu in PM muscle. IL-6 targeted therapies such as tocilizumab might therefore also impact CD8^+^NKG2D^high^ cell mediated pathology in PM.

Cocultivation of naïve CD8^+^ T cells with IL15-expressing myoblasts led to an increased expression of activation markers, inflammatory cytokines and effector molecules (NKG2D, granzyme B, perforin) resulting in enhanced cytolotyic capabilities resembling the findings with cell-independent IL-15 stimulation. However, IL-15 was not detected in cell culture supernatants of myoblasts supporting previous reports suggesting cell membrane rather than secreted IL-15 as being crucial in IL-15 mediated *in vivo* effects [39]. In agreement, we detected IL-15 in PM biopsy specimens mainly bound to the cell membrane of muscle cells and to lesser extents in their cytoplasm. CD4^+^ T cells and CD68^+^ macrophages were identified as yet unknown additional sources of IL-15 in PM muscle. In contrast, a study by Zong [42] demonstrated IL-15 expression in PM muscle by CD163^+^ macrophages and myoblasts, but not by fully differentiated myotubes, whereas Sugiura [41] reported expression of IL-15 predominantly in the cytoplasm of muscle cells and to lesser extents by infiltrating mononuclear cells derived from PM patients. These discrepancies might be explained by different antibodies, staining protocols and patient selection.

Membrane-bound muscle cell IL-15 was able to induce CD8^+^NKG2D^high^ T cells *in vitro* and might therefore contribute to the generation of CD8^+^NKG2D^+^ positive T cells in the inflamed muscle of PM patients as indicated by highly prevalent CD8 and NKG2D double positive cells in PM biopsy specimens. Moreover, CD8^+^NKG2D^high^ T cell frequencies were increased in the peripheral blood of PM patients correlating with serum CK levels - as surrogate marker for disease activity - further arguing for a meaningful pathogenic role of this subset in PM. Hence, our data provide evidence for a functional implication of surface IL-15 in the synaptic interaction between CD8^+^ T cells and MHC class I expressing muscle cells characteristic for PM and IBM and in the generation of muscle-resident CD8^+^NKG2D^high^ T cells [40]. In agreement with a pathogenic relevance of IL-15, previous studies have suggested a correlation of IL-15 expression levels with disease activity parameters [42, 46] and treatment response [42] in inflammatory myopathies.

Of note, IL-15 has also been proposed as local factor in patients with multiple sclerosis (MS) maintaining autoimmune conditions in the CNS. IL-15 was produced by astrocytes and infiltrating macrophages in MS patients displaying increased IL-15 levels in the serum and cerebrospinal fluid. IL-15 attracted CD4^+^CD28^null^ and CD8^+^ T cells to the CNS enhancing NKG2D expression and other pathogenic effector mechanisms [47, 48]. However, IL-15 is not only able to attract, but also to generate CD28^null^ T cells [36, 37], the predominating subset of muscle-resident CD4^+^ and CD8^+^ T cells in inflammatory myopathies [5-7]. In our study around 50% of *in vitro* IL-15 generated CD8^+^NKG2D^high^ T cells were negative for CD28. Therefore, IL-15 might significantly contribute to the high frequencies of CD28^null^ T cells in inflammatory myopathies and be a valuable therapeutic target. However, half of the CD8^+^NKG2D^high^ T cells were still CD28 positive arguing for CD8^+^NKG2D^high^ T cells as a heterogeneous yet distinct cell subset.

The importance of the local inflammatory milieu might explain the lack of differences in serum sMICA levels comparing PM patients and healthy donors. Moreover, this might be – at least in part - attributed to the immunosuppressive therapy of PM patients.

**Figure 5:**
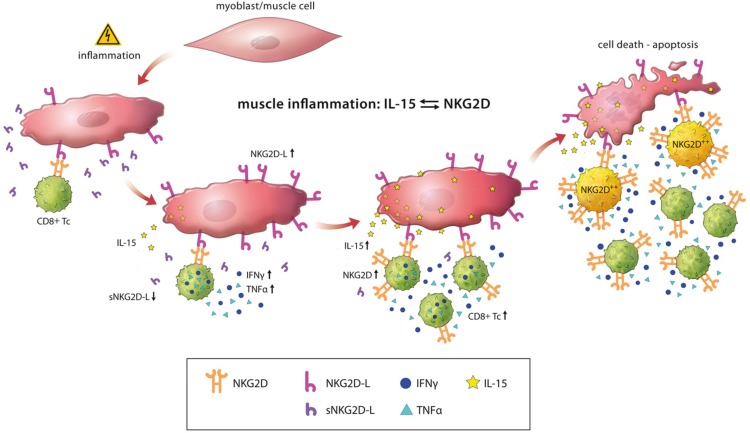
Schematic overview of NKG2D-mediated pathology in inflammatory myopathies Under inflammatory conditions, muscle cells show an upregulated surface expression of NKG2D ligands (NKG2D-L). CD8^+^NKG2D^high^ T cells (NKG2D^++^) are generated by the simultaneous reduction of soluble NKG2D ligands (sNKG2D-L) decreasing NKG2D internalization in CD8^+^ T cells together with IL-15 (predominantly membrane-associated) mediated stimulation by inflamed muscle cells. NKG2D-ligand engagement of CD8^+^NKG2D^high^ cells promotes IFNγ and TNFα production perpetuating the pro-inflammatory muscle environment and induces NKG2D-dependent/TCR-independent lysis of muscle cells. Of note, muscle-infiltrating CD68^+^ macrophages and CD4^+^ T cells might be further sources of IL-15 (not depicted).

Taken together, we here propose a pathological model, wherein muscle cells start to express NKG2D ligands and IL-15 in an already ongoing autoimmune inflammation or *de novo* upon an unknown inflammatory or environmental factor. Interaction with CD8^+^ T cells leads to the generation of highly pathogenic CD8^+^NKG2D^high^ T cells maintaining the local inflammatory milieu ultimately leading to muscle cell death (Figure 5). IL-15 and NKG2D as well as their cognate ligands might be important in stabilizing the immunological synapse between CD8^+^ T cells and MHC class I expressing muscle cells in idiopathic inflammatory myopathies. Future studies using experimental models and therapeutic interventions might unravel the underlying pathological pathways and the potential of targeting NKG2D – IL-15 signaling in inflammatory myopathies.

## MATERIALS AND METHODS

### Clinical samples

Tissue specimens from patients with polymyositis (PM, n = 9) and non-myopathic control subjects (healthy donors, HD, n = 6) were obtained as described before [49]. All samples were snap-frozen within 5 min after surgical intervention and stored at −80°C until analysis. The diagnosis was based on the patient's medical history, clinical and histopathological features according to the criteria of the European Neuromuscular Centre (ENMC) [50].

Peripheral blood and serum was sampled from PM patients (n = 10 / n = 5) and sex- and age-matched healthy donors (n = 13 / n = 5) following standard protocols. For PM patients the median age was 57 years (range 30 – 65) and median disease duration was 11 months (range 3 – 144). At time of blood sampling patients were treated with prednisolone alone (n = 3), methotrexate (n = 1), methotrexate in combination with intravenous immunoglobulins (n = 1), azathioprine (n = 2), cyclophosphamide (n = 1) and intravenous immunoglobulins (n = 2). For individual patient characteristics, see Table 1.

All patients and healthy donors gave informed consent according to the Declaration of Helsinki and the study was approved by the ethics committee of the University of Münster (AZ 2014-399-f-S).

### Antibodies and reagents

The following primary anti-human antibodies were used for flow cytometry, the respective isotype controls were purchased from BD Biosciences: CD3-BV510^3^, CD4-APC^5^, CD8-PerCP^1^, CD8-PB^2^, CD14-BV510^3^, CD19-APC^1^, CD25-FITC^1^, CD25-APC^5^, CD28-FITC^4^, CD44-FITC^1^, CD56-APC^1^, CD62L-APC^1^, CD69-APC^1^, CD107a-FITC^1^, CD244-PerCP-Cy5.5^3^, granzyme B-AF700^1^, FAS-L-PE^3^, IFNγ-FITC^1^, IFNγ-AF700^3^, IL-15-APC^6^, MHC-I-FITC^3^, MICA/B-PE^1^, NKG2D-PE^7^, perforin-PE^3^, TNFα-PE^3^, TCRγδ-PerCP-Cy5.5^3^, ULBP-1^6*^, ULBP-2-APC^6^, ULBP-3^6*^, (*Secondary labeled with goat anti-mouse-PE, Sigma-Aldrich; ^1^BD Biosciences; ^2^Beckman Coulter; ^3^Biolegend; ^4^eBioscience; ^5^Miltenyi Biotec; ^6^R&D).

Blocking humanized anti-NKG2D antibody was obtained from Novo Nordisk. The corresponding isotype was human IgG4, kappa (Sigma-Aldrich). Blocking IL-15 antibody and the corresponding isotype control were purchased from R&D Systems and used as described before [48].

Human interferon (IFN)-γ and tumor necrosis factor (TNF)-α were produced by Peprotech. Anti-human CD3 (OKT-3, 1 mg/ml; University of Mainz), human IL-6 and IL-15 (both Peprotech) were used for T cell activation.

Carboxyfluorescein succinimidyl ester (CFSE) and DiD (1,1′-dioctadecyl-3,3,3′,3′-tetramethylindodicarbocyanine) were both purchased from Invitrogen.

### Cell isolation and cell culture

Peripheral blood mononuclear cells (PBMCs) were isolated from healthy donors and PM patients by centrifugation on a Lymphoprep™ (Fresenius Kabi Norge AS) density gradient. CD8^+^ T cells were negatively purified using the CD8^+^ T cell Isolation Kit (Miltenyi Biotec) following the manufacturer's instructions. Cell purity was analyzed by flow cytometry and revealed > 95% of CD8^+^ cells. CD8^+^ T cells were cultured in RPMI supplemented with 10% FCS, 1% L-glutamine and 1% penicillin/streptomycin.

Primary human myoblasts were isolated from diagnostic muscle biopsy specimens as previously described [10]. Proliferating myoblasts were further purified by NCAM (neural cell adhesion molecule, CD56, clone 5.1H11, hybridoma supernatant) magnetic bead separation. The purity of myoblast cultures was assessed by flow cytometry and showed > 95% NCAM positive cells (Suppl. Figure 1). Human myoblasts were cultured in skeletal muscle cell growth medium (Promocell) at 37°C, 5% CO_2_.

### Flow cytometry

Flow cytometric analysis of PBMCs was performed following standard protocols. Cells were analyzed on a BD FACS Calibur Flow Cytometer (BD Biosciences) or a Gallios Flow Cytometer (Beckman Coulter).

For intracellular cytokine staining, the BD Cytofix/Cytoperm™ Fixation/Permeabilization Solution Kit was used (BD Biosciences) according to the manufacturer's instructions. In one set of experiments CD8^+^ T cells were isolated after coculture with myoblasts and restimulated with PMA (50 ng/ml) and ionomycin (1 μg/ml) for 4 h with BD GolgiPlug™ (BD Biosciences) prior to intracellular cytokine staining.

### RT-PCR

We isolated RNA from human myoblasts following standard procedures. We performed cDNA synthesis using a standard protocol with random hexamer primers (Applied Biosystems) and run RT-PCR with FAM labeled Taqman primers (all from Applied Biosystems) for ADAM9 (Hs00177638_m1), ADAM10 (Hs00153853_m1), ADAM17 (Hs01041915_m1), DAP10 (HCST, Hs00367159_m1) and VIC labeled 18S rRNA as endogenous control. We performed RT-PCR for 35 cycles and measured samples as triplicates. We calculated the data using the change in cycle threshold (ΔCT), ΔΔCT and relative quantification (2^−ΔΔCT^).

### FATAL assay (fluorometric assessment of T lymphocyte antigen specific lysis)

The assay was performed with modifications as described before [45]. In brief, 1×10^6^ primary human myoblasts were labelled with DiD (membrane dye, 5×10^−5^ M) followed by CSFE staining (cytosolic dye, 2.5×10^−6^ M) according to the manufacturer's instructions and inflamed for 48 h with IFNγ (1000U/ml) and TNFα (1000U/ml). Human leukocyte antigen (HLA)-A2 mismatched, alloreactive CD8^+^ T cells were stimulated with coated anti-CD3 (1 μg/ml) for 24 h followed by IL-15 (20 ng/ml) for 8 d to generate CD8^+^NKG2D^high^ cells as described before [20]. Afterwards CD8^+^NKG2D^high^ cells were cocultured with DiD/CFSE stained primary human myoblasts for 5 h at 37°C, 5% CO_2_. Blocking anti-NKG2D (10 μg/ml) or the respective isotype control (10 μg/ml) was added to the coculture 4 h prior and during the assay. The effector/target ratios (E/T) were 20/1 and 40/1. Cells were harvested and analyzed by flow cytometry on a FACSCalibur™ (BD Biosciences). The loss of CFSE in DiD^+^ myoblasts represents the proportion of lysed cells.

### ELISA

Detection of soluble MICA: the supernatants of myoblast cell cultures were analyzed for the presence of MICA by ELISA as described before [51].

In another set of experiments IFNγ, TNFα and IL-6 were determined by ELISA in cell culture supernatants of IL-15 stimulated CD8^+^ T cells following standard protocols.

### Immunofluorescence/immunohistochemistry

For MICA/B staining 8-10 μm sections of muscle biopsy specimens were fixed with PFA (4%) for 10 min at room temperature, washed three times with PBS and incubated with 5% BSA (PAA), 1% normal goat-serum (PAA) and 0.2% Triton-X100 (Sigma-Aldrich) for 1 h at room temperature. Afterwards, sections were incubated with the primary antibody anti-human MICA/B (R&D Systems, 1:100) overnight at 4°C. Sections were then stained with the secondary antibody goat anti-mouse Cy3, (Dianova, 1:200) for 1 h at room temperature. Next, after washing steps, sections were mounted with Prolong Gold antifade reagent with DAPI (Life Technologies), covered and analyzed on an Axio Scope.A1 fluorescence microscope (Zeiss).

For double-immunofluorescence staining 8-10 μm sections of muscle biopsy specimens were adapted to room temperature (RT) for 20 min and fixed with acetone for 10 min. Afterwards the sections were incubated with the blocking agent (goat serum 1:10 in PBS) for 30 min at RT. Followed by incubation with the primary antibodies: anti-human IL-15 (Abcam, 1:100) or anti-human NKG2D (Abcam, 1:75) overnight at 4°C. Sections were then stained with the secondary antibodies (goat anti-rabbit Cy3 or goat anti-mouse Cy3, Dianova, 1:100) for 1 h at room temperature. After a washing step a second blocking was performed for 10 min at RT. In the next step the second primary antibody was applied for 1 h at RT (anti-human CD4 (Zytomed, ready to use), anti-human CD8 (DAKO, 1:100) or anti-human CD68 (DAKO, 1:1000); followed by the second secondary antibodies (goat anti-rabbit AF488 or goat anti-mouse AF488, Dianova, 1:100). After a final washing step, sections were mounted with VECTASHIELD® Mounting Medium with DAPI (Vector), covered and analyzed on a Zeiss Observer.Z1 Microscope with the Axiovision 4 Software (Zeiss, Germany).

Further 8-10 μm sections of muscle biopsy specimens used for immunohistochemistry were fixed with acetone for 10 min, air-dried and incubated with goat serum (1:10 in PBS) for 30 min at room temperature. Afterwards, sections were incubated with the primary antibodies: anti-human IL-15 (Abcam, 1:100), anti-human NKG2D (Abcam, 1:75) overnight at 4°C. After washing with PBS, sections were stained with the secondary antibody (goat anti-rabbit or goat anti-mouse HRP (horseradish peroxidase)-conjugated, Dianova, 1:100) for 1 h at RT. After the next washing steps, 20 μl of DAB chromogen were added to 1 ml of DAB substrate (both from Dako), mixed and applied to the tissue. After incubation, reaction was stopped with distilled water. Then sections were counterstained with haemalum, dehydrated with alcohol, covered and analyzed on an Olympus BX50 microscope, with the digital camera DP25 and the Cell^D software (Olympus, Germany).

In another set of experiments slices were stained with hematoxylin/eosin to visualize immune cell infiltration according to standard protocols.

### Statistical analysis

All results are presented as mean ± SEM. Statistical analysis was performed using a modified Student's *t*-test in case of normally distributed data, or a Mann-Whitney test for parametric data without normality and equality of variance and for non-parametric datasets. A one-way ANOVA with Bonferroni post hoc test was used in the case of multiple comparisons for parametric data, and a Kruskal-Wallis ANOVA was used for non-parametric data. P values < 0.05 were considered statistically significant.

## SUPPLEMENTARY MATERIAL AND FIGURES


