# Supplementary material for: The NKG2D – IL-15 signaling pathway contributes to T-cell mediated pathology in inflammatory myopathies
Source: Oncotarget. 2015 Dec 4;6(41):43230–43. doi: 10.18632/oncotarget.6462 (PMC4791228; doi:10.18632/oncotarget.6462)
Supplement: Supplementary file 1 [file oncotarget-06-43230-s001.pdf]

The NKG2D – IL-15 signaling pathway contributes to T-cell mediated pathology in inflammatory myopathies

Supplementary Material

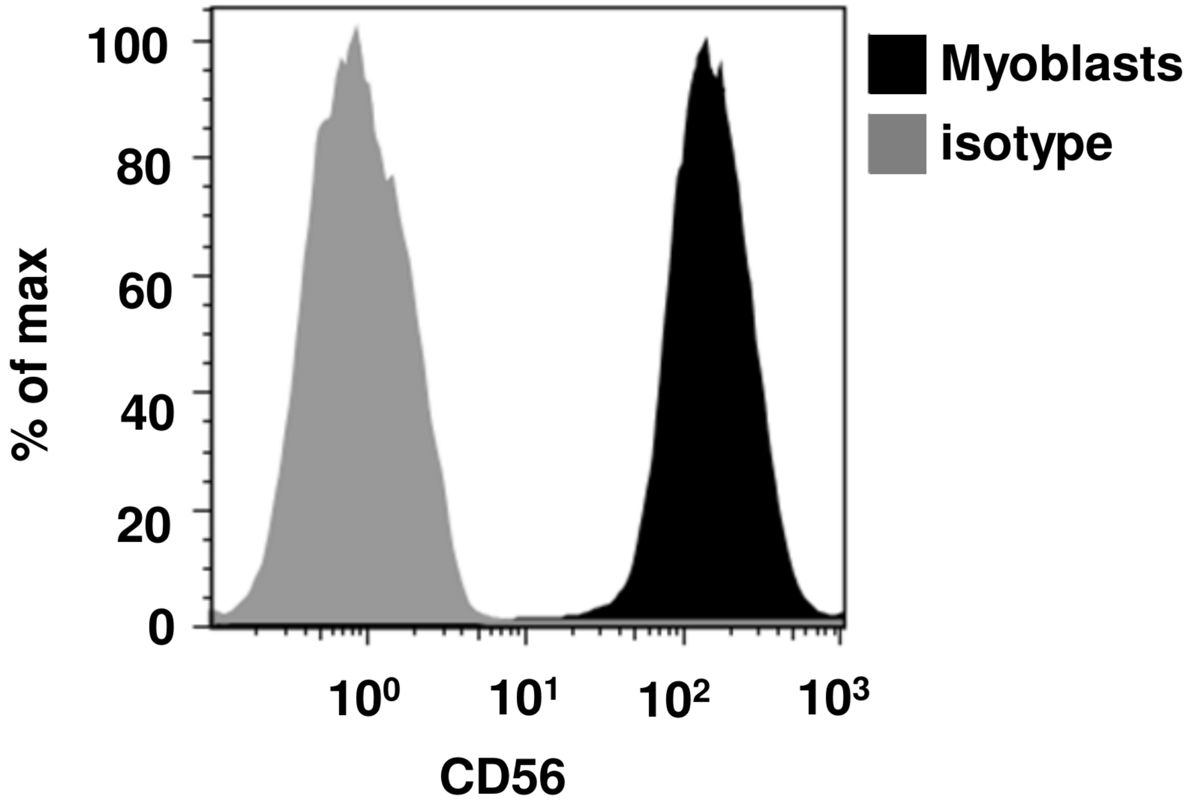

Supp. Fig. 1 Purity of primary human myoblast cell cultures. Flow cytometry analysis for CD56 expression of myoblasts compared to isotype control, >98% of cells were $CD56^+$.

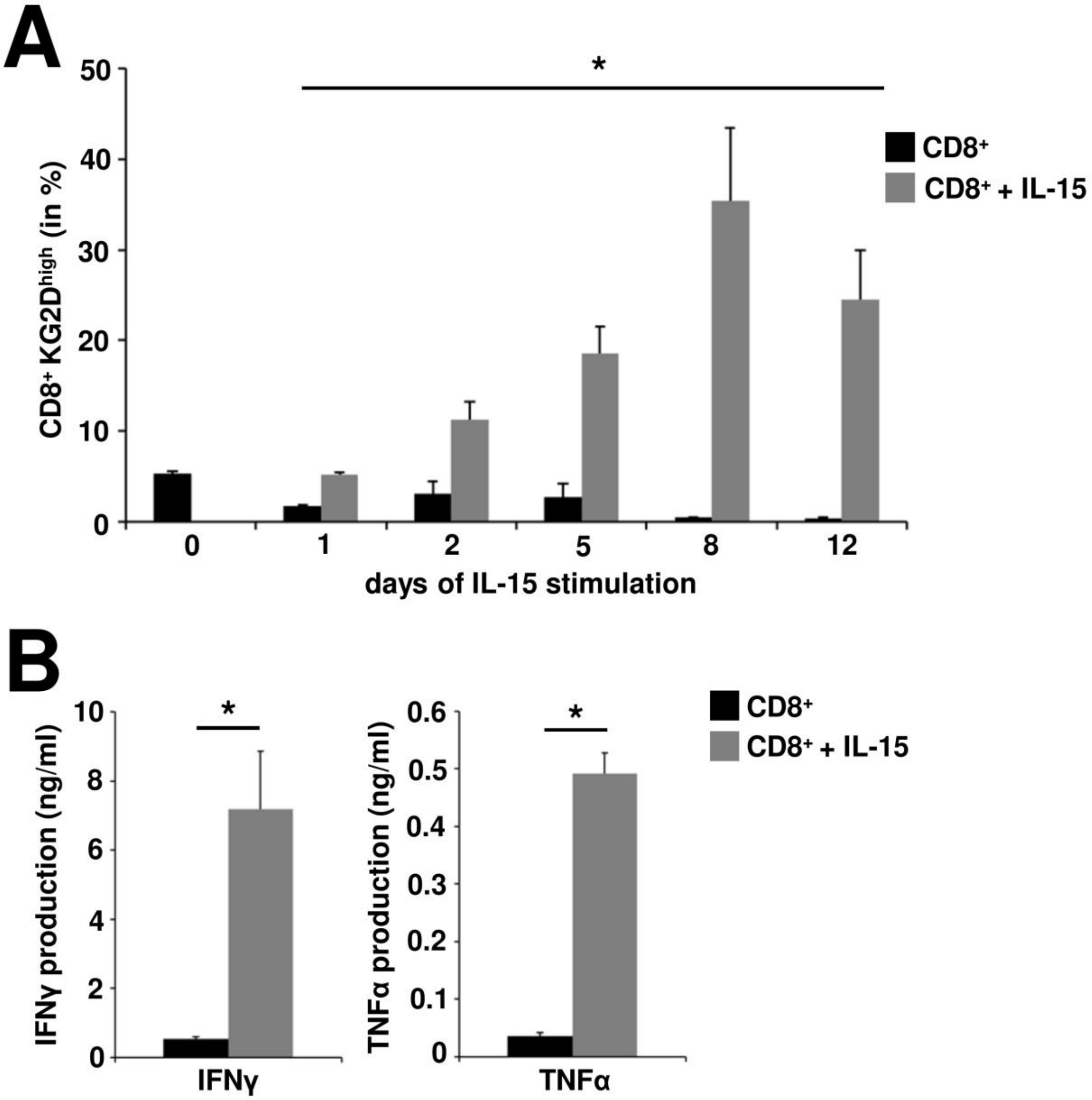

Supp. Fig. 2 CD8^{+}NKG2D^{high} cells are generated by IL-15 and produce high amounts of proinflammatory cytokines. (**A**) Frequencies of CD8^{+}NKG2D^{high} cells under chronic IL-15 stimulation obtained by flow cytometry (n = 3). (**B**) IFNγ and TNFα production of CD8^{+}NKG2D^{high} cells compared to CD8^{+} T cells under control conditions assessed by ELISA (n = 7).

* $p < 0.05$

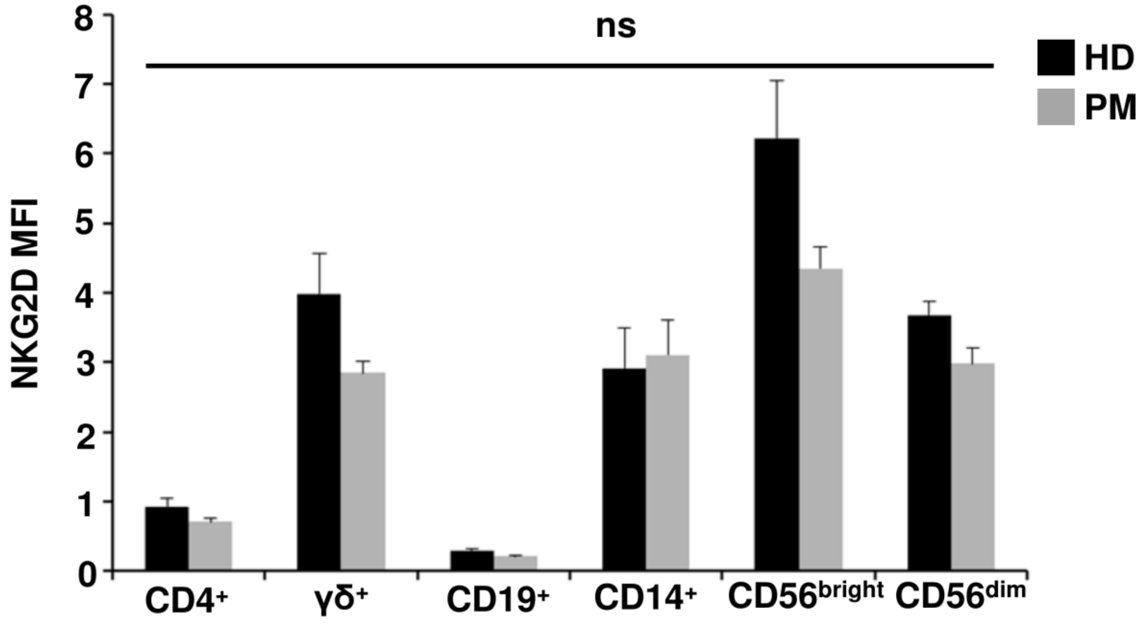

Supp. Fig. 3 No differences in NKG2D expression of CD4$^+$ T cells, γδ cells, B cells, NK cells and monocytes on PBMCs of HD and PM patients. Flow cytometry analysis of peripheral blood mononuclear cells (PBMCs) from healthy donors (HD, n = 13) and polymyositis patients (PM, n = 10) stained for different lineage markers and NKG2D.
